# Synovial-to-blood glucose ratio as a biomarker for the early diagnosis of septic arthritis of the knee in emergency medical practice: a retrospective clinical cohort study

**DOI:** 10.1186/s12891-025-09297-1

**Published:** 2026-01-12

**Authors:** Hiroshi Okuno, Shinjiro Miura, Kiyotsugu Shinagawa, Tomonori Kunii, Daisuke Chiba, Hiroaki Ogawa, Shingo Nobuta, Toshimi Aizawa, Eiji Itoi

**Affiliations:** 1 Department of Orthopaedic Surgery, Tohoku Rosai Hospital, Sendai, Japan; 2 Department of Orthopaedic Surgery, Tohoku University Hospital, Sendai, Japan

**Keywords:** Diagnosis, Emergency department, Septic arthritis, Synovial-to-blood glucose ratio

## Abstract

**Background:**

This study aimed to analyze both hematological and synovial biomarkers in patients with septic arthritis (SA) and patients with non-septic arthritis (non-SA) of the knee and identify useful biomarkers for the early diagnosis of SA in emergency medical practice.

**Methods:**

We examined 397 knee joints of 367 patients with painful/swollen knee joints and potential infection at our department between May 2018 and May 2023. After excluding cases involving joints with prosthetic replacements, symptom duration more than 3 weeks, prior antibiotic use, and absence of arthrocentesis, 179 knee joints (166 patients) were assessed. To identify useful diagnostic parameters of the SA and non-SA groups, we compared their hematological and synovial fluid biomarkers and assessed the diagnostic performance of those biomarkers by conducting receiver-operating characteristic (ROC) analyses.

**Results:**

Hematological parameters, including the white blood cell (WBC) count, neutrophil sequestration, C-reactive protein level, and procalcitonin level, in the SA group were higher than those in the non-SA group. A synovial fluid examination revealed that the WBC count and neutrophil sequestration exhibited the same pattern. Synovial glucose levels and synovial-to-blood glucose ratios in the SA group were lower than those in the non-SA group. The ROC analysis revealed that the synovial-to-blood glucose ratio had the highest diagnostic utility for predicting SA.

**Conclusions:**

The synovial-to-blood glucose ratio appears to be a promising biomarker; however, its ability to differentiate SA from non-SA warrants further validation. The synovial-to-blood glucose ratio may complement existing diagnostic tools and improve early diagnostic precision in emergency medical settings.

## Background

 Acute pain and swelling in large joints have diverse etiologies, including trauma, infection, leukemia, crystal-induced arthritis, autoimmune diseases, osteonecrosis, and osteoarthritis [1]. Among these conditions, acute septic arthritis (SA) of the native knee in the emergency department (ED) represents a critical orthopedic emergency. Although the estimated global incidence of acute SA varies between 1 and 35 cases per 100,000 person-years [2], early identification and management of this rare condition are imperative. Administration of suitable antibiotics within the first 24–48 h of symptom onset is vital to preventing subchondral bone damage and permanent joint function impairment [3]. Despite appropriate antibiotic therapy, acute SA is associated with considerable morbidity and mortality in adults [4, 5]. Furthermore, delayed treatment significantly increases the risk of irreversible joint damage and death [4, 6].

Diagnosing acute SA can be challenging, even for experienced clinicians [7], because of the limited sensitivity (29%–65%) of synovial fluid Gram staining [8, 9] and the time required (24–48 h) for more sensitive synovial fluid cultures (75%–95%) to yield results [8]. Prior antibiotic exposure may further reduce the culture yield, resulting in greater difficulty determining an early diagnosis. Traditional biomarkers, including the C-reactive protein (CRP) level, erythrocyte sedimentation rate, and white blood cell (WBC) count, are frequently used because of their affordability and rapid availability. However, these markers have good sensitivity but poor specificity for precisely diagnosing infectious conditions [10, 11]. Therefore, efficient and accessible diagnostic tools that allow rapid identification of SA in emergency medical settings are necessary.

Recent evidence has highlighted the diagnostic potential of synovial glucose levels for SA [12, 13]. Synovial glucose levels are usually within 10–20 mg/dL (approximately 0.6–1.1 mmol/L) of the fasting blood glucose level [14]; however, in the context of SA, synovial glucose levels are further reduced [12, 13]. Previous studies have also evaluated the intrasynovial lactate-to-glucose ratio [12], which is distinct from the synovial-to-blood glucose ratio. The synovial-to-blood glucose ratio may be more reliable than the intrasynovial lactate-to-glucose ratio because it accounts for systemic hyperglycemia, particularly in patients with diabetes; however, its diagnostic utility has not been firmly established. Although our cohort comprised adult patients, we referred to some studies that included pediatric populations to review general principles.

This study assessed hematological and synovial biomarkers in patients with and without SA and focused on the diagnostic utility of the synovial-to-blood glucose ratio for the early detection of SA in emergency medical practice.

## Methods

This retrospective clinical cohort study included 397 knee joints of 367 patients who had painful/swollen knee joints and potential infection. These patients presented to our institute between May 2018 and May 2023. The exclusion criteria were as follows: absence of arthrocentesis; more than 3 weeks between symptom onset and the initial visit; prior prosthetic replacement of the knee; and prior antibiotic use before the initial visit. After applying these criteria, 179 knee joints of 166 patients were analyzed.

The following were used to define SA: the presence of neutrophils with phagocytosed bacteria following Gram staining of synovial fluid; identification of bacteria in synovial fluid cultures; or identification of bacteria in blood cultures of patients with purulent synovial fluid and clinical features of SA, including systemic symptoms such as fever, chills, and general fatigue as well as local findings such as knee joint pain, swelling, heat, and redness. However, Gram staining has limited sensitivity and culture results may be affected by prior antibiotic use, which could lead to misclassifications.

Non-SA included pseudo-gouty arthritis, gouty arthritis, rheumatic arthritis, osteoarthritis, and traumatic arthritis. Pseudo-gout and gout were differentiated based on the identification of calcium pyrophosphate and sodium urate crystals, respectively, in the synovial fluid and failure to meet the diagnostic criteria for SA. Rheumatic arthritis was defined as the presence of cloudy joint fluid accompanied by rheumatic diseases, including rheumatoid arthritis, psoriatic arthritis, other types of spondyloarthritis, and failure to fulfill the diagnostic criteria for SA and crystal-induced arthritis. Osteoarthritis was defined as a Kellgren–Lawrence grade ≥ 1 [15] according to radiography and failure to fulfill the diagnostic criteria for SA, crystal-induced arthritis, and rheumatic arthritis. Traumatic arthritis was defined as a condition involving a history of trauma and clinical symptoms similar to those of SA.

### Measurements

At the initial visit, comprehensive assessments of all patients, including a medical interview, palpation, blood collection, sterile joint aspiration, radiographic evaluation to determine the diagnosis, and subsequent treatment, were conducted. Quantification of biomarkers in the blood (WBC count, neutrophil sequestration, procalcitonin level, CRP level, and glucose level) and synovial fluid (WBC count, segmented neutrophil sequestration, and glucose level) was performed at our central laboratory.

Aspirated synovial fluid samples were immediately divided into three tubes for the bacterial culture, crystal identification, and hematological examination. These tubes contained EDTA-2K (Venoject II; Terumo, Tokyo, Japan) and were used to investigate the WBC count, WBC fraction, and glucose level in synovial fluid. Synovial fluid in the hematological examination tube was supplemented with 100 µL of 0.2% hyaluronidase (Nacalai, Kyoto, Japan) before the analysis [16]. Synovial glucose was measured promptly after specimen preparation; however, when unavoidable delays occurred, samples were cooled and analyzed as soon as possible to minimize glycolysis. The erythrocyte sedimentation rate was not consistently measured; therefore, it was not included in the analysis.

### Identification of biomarkers

First, we compared the demographic characteristics of patients in the SA and non-SA groups. Second, we examined the results of the bacteria smear culture and analyzed the causative bacteria in the SA group. Third, to identify factors that were useful for differentiating between the SA and non-SA groups, we compared their biomarkers in the blood and synovial fluid. Finally, a receiver-operating characteristic (ROC) analysis of biomarkers with *P* < 0.05 in the univariate analysis was performed. The ROC curve and area under the ROC curve (AUC) were analyzed to assess the sensitivity, specificity, positive predictive value, and negative predictive value for the diagnosis of SA. The optimal cutoff value was defined as the point on the curve closest to (0, 1) [17]. The AUC values are presented with 95% confidence intervals (CIs). However, our study was limited because we did not perform multivariable logistic regression, multiple comparison correction, or internal validation (e.g., bootstrap).

### Statistical analysis

All statistical analyses were performed using GraphPad Prism version 8 (GraphPad Prism Software Inc., San Diego, CA, USA) and JMP Pro 17.0 (SAS Institute Inc., Cary, NC, USA). Between-group comparisons of age, interval between symptom onset and the initial visit, body temperature at the initial visit, blood biomarkers, and synovial fluid biomarkers were performed using the Mann–Whitney U test. Fisher’s exact test was used to compare the sex distribution, type of affected joints, unilateral-to-bilateral ratio, type of arthroplasty, and comorbidities. Statistical significance was set at *P* < 0.05.

### Ethics, registration, data sharing plan, funding, and disclosures

This study was performed in accordance with the Declaration of Helsinki and reviewed and approved by the Institutional Review Board of our institute (No. 23 − 9). Informed consent was obtained in the form of an opt-out option on our website.

## Results

### Patient demographics

Among 397 knee joints of 367 patients with painful/swollen knee joints, 218 knee joints of 201 patients were excluded according to the predefined criteria. Ultimately, 179 knee joints of 166 patients were included in this study and divided into two groups. One group comprised 20 knee joints of 20 patients with SA (SA group) and the other comprised 159 knee joints of 146 patients with non-SA (non-SA group). Because of the small number of SA cases, the estimates presented in this work may be imprecise.

Patient demographics are shown in Table 1. No significant between-group differences in sex, age, unilateral-to-bilateral ratio, interval between symptom onset and the initial visit, body temperature at the initial visit, or comorbidities such as rheumatoid arthritis and diabetes mellitus were observed. However, because of the small number of SA cases, the possibility of a type II error cannot be excluded.

**Table 1:** Demographics of the patients

Characteristics	Septic arthritis (*n* = 20 knees of 20 patients)	Nonseptic arthritis (*n* = 159 knees of 146 patients)	*P*
Sex			0.310
Male	9 (45.0%)	46 (32.4%)	
Female	11 (55.0%)	100 (67.6%)	
Age, years (range)	69 (28 − 93)	75 (31 − 100)	0.077
Unilateral : bilateral	20 : 0	133 : 13	0.370
Median interval between symptom onset and initial visit, days (range)	4.5 (0 − 14)	5.5 (0 − 21)	0.853
Mean body temperature at the initial visit, °C (range)	37.3 (36.2 − 39.2)	37.5 (36.0 − 39.9)	0.458
Comorbidity			
Rheumatoid arthritis	4 (20.0%)	27 (18.2%)	> 0.999
Diabetes mellitus	2 (10.0%)	12 (8.1%)	0.678
Diagnosis			
Septic arthritis	20	0	
Nonseptic arthritis	0	146	
Pseudo-gout	0	66 (45.2%)	
Gout	0	10 (6.8%)	
Rheumatic arthritis	0	20 (13.7%)	
Osteoarthritis	0	37 (25.3%)	
Trauma	0	6 (3.8%)	
Others	0	7 (4.8%)	

The values are those of the patients. The number of affected knees is provided in parentheses when relevant

### Microorganisms identified in the SA group

**Fig. 1:**
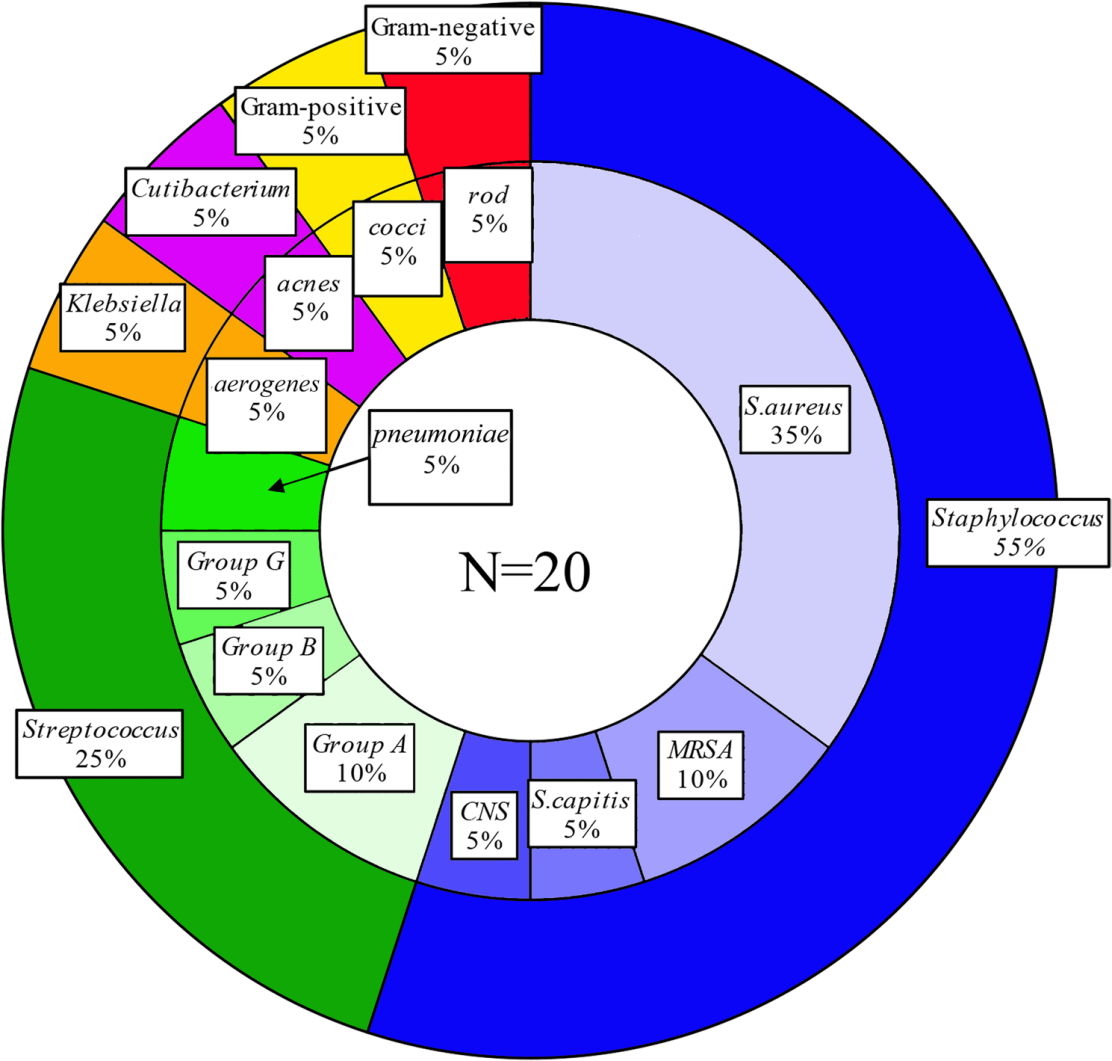
Distribution of microorganisms identified in patients with septic arthritis (SA) by the bacterial culture. *Staphylococcus* species were identified most often, followed by *Streptococcus pneumoniae*. CNS, coagulase-negative *Staphylococcus*; MRSA, methicillin-resistant *Staphylococcus aureus*

Microorganisms in the SA group identified by the bacterial culture are shown in Table 2. Gram staining of 19 cases (95%) was performed, and 11 (58%) exhibited Gram-positive bacteria. Gram staining of two cases involving negative joint fluid cultures revealed neutrophils with phagocytosed bacteria. Based on our definition of SA, the sensitivities of the joint fluid culture and Gram staining were 90% and 58%, respectively. Microorganisms identified in patients with SA were most commonly *Staphylococcus s*pecies, followed by *Streptococcus pneumoniae* (Fig. 1). Because of the small number of SA cases, the distribution of organisms should be interpreted with caution.

**Table 2:** Microorganisms identified by the bacterial culture and neutrophil phagocytosis identified by gram staining in the septic arthritis group (20 knee joints of 20 patients)

**Bacteria**	Affected joints (*n*)	**Neutrophil phagocytosis (+) identified by Gram staining**	**Total (%)**
**Staphylococcus species (11; 55%)**			
S. aureus	7	4	35
MRSA	2	1	10
S. capitis	1	0	5
CNS	1	1	5
**Streptococcus species (5; 25%)**			
Group A	2	1	10
Group B	1	0	5
Group G	1	1	5
Streptococcus pneumoniae	1	1	5
**Klebsiella aerogenes**	1	0	5
**Cutibacterium acnes**	1	0	5
**Gram-positive cocci**	1	1	5
**Gram-negative rods**	1	1	5

*CNS* coagulase-negative *Staphylococcus*, *MRSA* methicillin-resistant *Staphylococcus aureus*

### Biomarkers in blood and synovial fluid

Table 3 presents the results of a comparative analysis of biomarker levels in the blood and synovial fluid of the SA and non-SA groups. Regarding blood biomarkers, the WBC count in the SA group (median, 11,900/µL; interquartile range [IQR], 8,000–17,100/µL]) was higher than that in the non-SA group (median, 7,900/µL; IQR, 6,000–10,300/µL). Additionally, the neutrophil proportion in the SA group (median, 86%; IQR, 77%–90%) was higher than that in the non-SA group (median 75%; IQR, 68%–82%). The serum procalcitonin level (median, 0.48 ng/mL [IQR, 0.08–1.74 ng/mL] vs. 0.09 ng/mL [IQR, 0.03–0.17]) and serum CRP level (median, 18.0 mg/dL [IQR 8.2–29.3 mg/dL] vs. 6.3 mg/dL [IQR 2.0–12.8]) in the SA group were significantly higher than those in the non-SA group.

**Table 3:** Biomarkers in blood and synovial fluid. Data are expressed as median (IQR). Several biomarkers had different numbers of analyzed samples; therefore, the column is labeled as “N (analyzed)”

**Biomarker**	**SA group (median, IQR)**	**N (analyzed)**	**Non-SA group (median, IQR)**	**N (analyzed)**	*P* value
Blood WBC (/µL)	11,900 (8000–17,100)	20	7900 (6000–10,300)	126	<0.001
Blood neutrophil (%)	87 (77–90)	20	75 (68–82)	123	<0.001
Serum procalcitonin (ng/mL)	0.48 (0.08–1.74)	12	0.09 (0.03–0.17)	52	0.001
Serum CRP (mg/dL)	18.0 (8.2–29.3)	20	6.3 (2.0–12.8)	126	<0.001
Synovial WBC (/µL)	71,000 (44,000–90,000)	13	18,000 (3500–29,000)	139	<0.001
Synovial neutrophil (%)	88 (85–92)	14	83 (60–90)	139	0.032
Synovial glucose (mg/dL)	13 (1–27)	11	97 (79–114)	118	<0.001
Synovial-to-blood glucose ratio	0.13 (0.01–0.25)	11	0.79 (0.62–0.89)	103	<0.001

Data are expressed as the median (IQR). Several biomarkers, such as blood WBC, blood neutrophil proportion, serum procalcitonin, serum CRP, synovial WBC, synovial neutrophil proportion, synovial glucose, and synovial-to-blood glucose ratio, were missing values

*CRP* C-reactive protein, *IQR* interquartile range, *SA* septic arthritis, *WBC* white blood cell

Regarding synovial biomarkers, the WBC count (median, 71,000/µL [IQR, 44,000–90,000/µL] vs. 18,000/µL [IQR, 3,500–29,000/µL]) and segmented neutrophil proportion (median, 88% [IQR, 85%–92%] vs. 83% [IQR, 60%–90%]) in the SA group were significantly higher than those in the non-SA group. In contrast, the synovial glucose concentration (median, 13 mg/dL [IQR, 1–27 mg/dL] vs. 97 mg/dL [IQR, 79–114 mg/dL]) and synovial-to-blood glucose ratio (median, 0.13 [IQR, 0.01–0.25] vs. 0.79 [IQR, 0.62–0.89]) in the SA group were significantly lower than those in the non-SA group.

#### Diagnostic potential of the synovial-to-blood glucose ratio

Table 4 shows the results of the ROC curve analysis of the investigated parameters for diagnosing SA. The ROC analysis revealed that a synovial-to-blood glucose ratio < 0.55 had the highest diagnostic potential for predicting SA (AUC, 0.975; 95% CI, 0.95–1.00).

**Table 4:** ROC analysis of the parameters for the diagnosis of septic arthritis in patients with painful/swollen knees who visited our institute between May 2018 and May 2023

	Blood WBC count (/µL)	Blood neutrophil sequestration (%)	Serum procalcitonin (ng/dL)	Serum CRP (mg/dL)	Synovial WBC count (/µL)	Synovial segmented neutrophil sequestration (%)	Synovial glucose (mg/dL)	Synovial-to-blood glucose ratio
AUC (95% CI)	0.73 (0.59–0.87)	0.76 (0.65–0.88)	0.79 (0.64–0.94)	0.76 (0.64–0.88)	0.86 (0.74–0.98)	0.67 (0.56–0.78)	0.972 (0.94–1.0)	0.975 (0.95–1.0)
Cutoff	9,650	83.9	0.38	11.8	42,400	83.8	57.5	0.55
Sensitivity (95% CI)	0.70 (0.48–0.85)	0.60 (0.39–0.78)	0.67 (0.39–0.86)	0.75 (0.53–0.89)	0.84 (0.58–0.97)	0.93 (0.69–1.0)	1 (0.74–1.0)	1 (0.74–1.0)
Specificity (95% CI)	0.71 (0.63–0.79)	0.83 (0.75–0.89)	0.92 (0.81–0.97)	0.71 (0.63–0.79)	0.88 (0.82–0.93)	0.50 (0.42–0.59)	0.89 (0.82–0.93)	0.88 (0.81–0.93)
PPV	0.28	0.36	0.67	0.29	0.41	0.16	0.46	0.48
NPV	0.94	0.93	0.92	0.95	0.98	0.99	1	1
N	146	143	64	146	152	153	129	114
ROC curve								

*AUC* area under the receiver-operating characteristic curve, *CI* confidence interval, *CRP* C-reactive protein, *N* number, *NPV* negative predictive value, *PPV* positive predictive value, *ROC* receiver-operating characteristic, *WBC* white blood cell

## Discussion

We conducted a comprehensive evaluation of clinical biomarkers in patients in the SA and non-SA groups in the ED setting to identify markers that could facilitate an early diagnosis. Our analysis demonstrated that the synovial-to-blood glucose ratio appeared to provide the best diagnostic performance in this cohort; however, prospective validation is required.

Traditional diagnostic methods for SA often involve waiting for culture results, which can delay treatment initiation. Although Gram staining provides rapid results, its sensitivity is low and prior antibiotic exposure may reduce the culture yield. In our cohort, *Staphylococcus aureus* was the most common organism, consistent with the findings of previous studies [18].

Among routinely available biomarkers, the synovial WBC count and serum CRP level are widely used; however, their specificity is limited. Synovial α-defensin has high accuracy [19, 20], but its use is limited by cost and sample requirements. In contrast, the synovial-to-blood glucose ratio is inexpensive and can be measured quickly without specialized reagents. In this study, the synovial-to-blood glucose ratio outperformed both the CRP level (AUC, 0.76; specificity, 0.71) and synovial WBC count (AUC, 0.86; specificity, 0.88), suggesting its potential value as a complementary diagnostic tool.

Prior research has also highlighted the diagnostic utility and feasibility of synovial glucose itself. Omar et al. [21] reported that synovial glucose measured by both a bedside glucometer and an automated analyzer exhibited high diagnostic accuracy for bacterial arthritis (AUC, 0.96), with a cutoff value of 1.4 mmol/L yielding 100% sensitivity and 92% specificity. Moreover, Kinugasa et al. [22] used a portable glucometer to obtain rapid synovial glucose measurements and found that a synovial glucose level < 40 mg/dL differentiated septic arthritis from transient synovitis. Although these data support the practicality and accuracy of synovial glucose—particularly in emergency medical settings—the synovial-to-blood glucose ratio may further complement such approaches by partly adjusting for systemic hyperglycemia (e.g., diabetes or stress-related hyperglycemia). Although we reviewed some studies that involved pediatric cohorts to examine general principles, our cohort only comprised adults. Furthermore, we used the synovial-to-blood glucose ratio because it is comparable to the cerebrospinal fluid-to-blood glucose ratio, which corrects for systemic hyperglycemia, for bacterial meningitis [23–27].

### Limitations

This retrospective single-center study had a small sample size, particularly that of the SA group (*n* = 20), which increased the risk of overestimation and unstable estimates. Because of the retrospective design, an a priori power calculation was not performed. The non-SA group was heterogeneous and included crystal-induced arthritis, rheumatic arthritis, and osteoarthritis; therefore, specificity was potentially diluted. Gram staining and cultures have imperfect sensitivity, and prior antibiotic exposure may have resulted in misclassifications. Additionally, we were unable to perform stratified analyses (e.g., patients with diabetes or those with crystal arthritis) because of the small sample size. This was an important limitation because systemic hyperglycemia and crystal-induced inflammation can influence the synovial-to-blood glucose ratio. Therefore, prospective multicenter studies that include larger cohorts are necessary to validate the synovial-to-blood glucose ratio among clinically relevant subgroups. Finally, we did not perform multivariable regression, multiple comparison correction, or internal validation, which further limited the generalizability of our findings. Additionally, we did not perform statistical comparisons between AUCs (e.g., DeLong test), which further limited the robustness of our findings.

### Practical applicability

The synovial-to-blood glucose ratio can be calculated rapidly in the ED by measuring blood and synovial glucose simultaneously, even with a portable glucometer. A cutoff value < 0.55 may result in high suspicion for SA and support early treatment decisions while awaiting confirmation from the culture results. This cutoff value may have been influenced by differences between measurement devices and the timing of sample acquisition; therefore, the results should be interpreted with caution.

## Conclusions

In summary, although our findings are preliminary, they suggest that the synovial-to-blood glucose ratio could be a useful and inexpensive biomarker that complements existing biomarkers for the early diagnosis of suspected SA in the ED. Additionally, it could be useful for differentiating SA from non-SA. Furthermore, this simple ratio could enable rapid diagnostic support in emergency medical settings while awaiting culture results. However, because of the retrospective and single-center design of this study as well as the limited number of SA cases, these results should be interpreted with caution. Prospective multicenter studies are warranted to validate our findings and determine the role of the synovial-to-blood glucose ratio in routine emergency medical practice, particularly for clinically relevant subgroups such as patients with diabetes and those with crystal-induced arthritis.

## Data Availability

The datasets used and/or analyzed during the current study are available from the corresponding author on reasonable request.

## Abbreviations

AUC: Area under the receiver-operating characteristic curve
CI: Confidence interval
CRP: C-reactive protein
CSF: Cerebrospinal fluid
IQR: Interquartile range
ROC: Receiver-operating characteristic
SA: Septic arthritis
WBC: White blood cell

## References

[1] Sack K. Monarthritis: differential diagnosis. Am J Med. 1997;102:S30–4. 10.1016/s0002-9343(97)00414-2 9217557

[2] He M, Arthur Vithran DT, Pan L, Zeng H, Yang G, Lu B, et al. An update on recent progress of the epidemiology, etiology, diagnosis, and treatment of acute septic arthritis: a review. Front Cell Infect Microbiol. 2023;13:1193645. 37249986 10.3389/fcimb.2023.1193645 PMC10214960

[3] Horowitz DL, Katzap E, Horowitz S, Barilla-LaBarca ML. Approach to septic arthritis. Am Fam Physician. 2011;84:653–60. 21916390

[4] Tarkowski A. Infection and musculoskeletal conditions: infectious arthritis. Best Pract Res Clin Rheumatol. 2006;20:1029–44. 17127195 10.1016/j.berh.2006.08.001

[5] Ferrand J, El Samad Y, Brunschweiler B, Grados F, Dehamchia-Rehailia N, Séjourne A, et al. Morbimortality in adult patients with septic arthritis: a three-year hospital-based study. BMC Infect Dis. 2016;16:239. 27246346 10.1186/s12879-016-1540-0 PMC4888402

[6] Abram SGF, Alvand A, Judge A, Beard DJ, Price AJ. Mortality and adverse joint outcomes following septic arthritis of the native knee: a longitudinal cohort study of patients receiving arthroscopic washout. Lancet Infect Dis. 2020;20:341–9. 31862240 10.1016/S1473-3099(19)30419-0

[7] Mathews CJ, Kingsley G, Field M, Jones A, Weston VC, Phillips M, et al. Management of septic arthritis: A systematic review. Ann Rheum Dis. 2007;66:440–5. 17223664 10.1136/ard.2006.058909 PMC1856038

[8] Carpenter CR, Schuur JD, Everett WW, Pines JM. Evidence-based diagnostics: adult septic arthritis. Acad Emerg Med. 2011;18:781–96. 21843213 10.1111/j.1553-2712.2011.01121.x PMC3229263

[9] Bram JT, Baldwin KD, Blumberg TJ. Gram stain is not clinically relevant in treatment of pediatric septic arthritis. J Pediatr Orthop. 2018;38:e536–40. 30036290 10.1097/BPO.0000000000001226

[10] Ben-Zvi L, Sebag D, Izhaki G, Katz E, Bernfeld B. Diagnosis and management of infectious arthritis in children. Curr Infect Dis Rep. 2019;21:23. 31144135 10.1007/s11908-019-0678-5

[11] Aggarwal P, Mahapatra S, Avasthi S, Aslam A, Kumar V. Role of serum and synovial procalcitonin in differentiating septic from non-septic arthritis- a prospective study. J Clin Orthop Trauma. 2022;31:101948. 35865327 10.1016/j.jcot.2022.101948 PMC9293762

[12] Berthoud O, Coiffier G, Albert JD, Gougeon-Jolivet A, Goussault C, Bendavid C, et al. Performance of a new rapid diagnostic test the lactate/glucose ratio of synovial fluid for the diagnosis of septic arthritis. Joint Bone Spine. 2020;87:343–50. 32234547 10.1016/j.jbspin.2020.03.009

[13] Lenski M, Scherer MA. Diagnostic potential of inflammatory markers in septic arthritis and periprosthetic joint infections: a clinical study with 719 patients. Infect Dis. 2015;47:399–409. 10.3109/00365548.2015.1006674 25746606

[14] Brannan SR, Jerrard DA. Synovial fluid analysis. J Emerg Med. 2006;30:331–9. 16677989 10.1016/j.jemermed.2005.05.029

[15] Kellgren JH, Lawrence JS. Radiological assessment of osteo-arthrosis. Ann Rheum Dis. 1957;16:494–502. 13498604 10.1136/ard.16.4.494 PMC1006995

[16] Hoshina H. Examination of pleural effusion, Ascites fluid and joint fluid. Kensa Gijutsu. 2014;42:1318–26.

[17] Akobeng AK. Understanding diagnostic tests 3: receiver operating characteristic curves. Acta Paediatr. 2007;96:644–7. 17376185 10.1111/j.1651-2227.2006.00178.x

[18] Roerdink RL, Huijbregts HJTAM, van Lieshout AWT, Dietvorst M, van der Zwaard BC. The difference between native septic arthritis and prosthetic joint infections: a review of literature. J Orthop Surg (Hong Kong). 2019;27:2309499019860468. 31284831 10.1177/2309499019860468

[19] Pupaibool J, Fulnecky EJ, Swords RL Jr., Sistrunk WW, Haddow AD. Alpha-defensin-novel synovial fluid biomarker for the diagnosis of periprosthetic joint infection. Int Orthop. 2016;40:2447–52. 27714447 10.1007/s00264-016-3306-0

[20] Bonanzinga T, Zahar A, Dütsch M, Lausmann C, Kendoff D, Gehrke T. How reliable is the alpha-defensin immunoassay test for diagnosing periprosthetic joint infection? A prospective study. Clin Orthop Relat Res. 2017;475:408–15. 27343056 10.1007/s11999-016-4906-0 PMC5213924

[21] Omar M, Reichling M, Liodakis E, Ettinger M, Guenther D, Decker S, et al. Rapid exclusion of bacterial arthritis using a glucometer. Clin Rheumatol. 2017;36:591–8. 27071629 10.1007/s10067-016-3255-4

[22] Kinugasa M, Kobayashi D, Satsuma S, Sakata R, Shinada Y, Kuroda R. The predictive value of synovial glucose level in septic arthritis. J Pediatr Orthop B. 2020;29:292–6. 30882559 10.1097/BPB.0000000000000628

[23] van de Beek D, de Gans J, Tunkel AR, Wijdicks EFM. Community-acquired bacterial meningitis in adults. N Engl J Med. 2006;354:44–53. 16394301 10.1056/NEJMra052116

[24] Julián-Jiménez A, Morales-Casado MI. Usefulness of blood and cerebrospinal fluid laboratory testing to predict bacterial meningitis in the emergency department. Neurología (English Edition). 2019;34:105–13. 10.1016/j.nrl.2016.05.009 27469578

[25] Rodewald LE, Woodin KA, Szilâgyi PG, Arvan DA, Raubertas RF, Powell KR. Relevance of common tests of cerebrospinal fluid in screening for bacterial meningitis. J Pediatr. 1991;119:363–9. 1880647 10.1016/s0022-3476(05)82046-3

[26] Viallon A, Desseigne N, Marjollet O, Birynczyk A, Belin M, Guyomarch S, et al. Meningitis in adult patients with a negative direct cerebrospinal fluid examination: value of cytochemical markers for differential diagnosis. Crit Care. 2011;15:R136. 21645387 10.1186/cc10254 PMC3219005

[27] Deisenhammer F, Bartos A, Egg R, Gilhus NE, Giovannoni G, Rauer S, et al. Guidelines on routine cerebrospinal fluid analysis. Report from an EFNS task force. Eur J Neurol. 2006;13:913–22. 16930354 10.1111/j.1468-1331.2006.01493.x

